# Utilization of smartphone application to resolve right-sided phrenic nerve stimulation for a pacemaker patient remotely located in rural farmland of Haiti

**DOI:** 10.1016/j.hroo.2022.10.003

**Published:** 2022-12-16

**Authors:** Rick Turek, Eric Toussaint, Omer Shedd

**Affiliations:** ∗ Cardiac Rhythm Management, Abbott Laboratories, Chicago, Illinois; † Hospital Bienfaisance, Pignon, Haiti; ‡ Promise for Haiti, Belmont, North Carolina

**Keywords:** Pacemaker, Phrenic nerve stimulation, Complete heart block, Smartphone application, Haiti

**Key Findings**

▪ Smartphone applications can be used to review, diagnose, and resolve medical challenges.
▪ Remote programming assistance via smartphone applications can reduce response time and improve patient experience and morbidity.
▪ Identification of medical issues can be spotted visually via smartphone apps.

*“Mr. Watson - come here – I want to see you.”* ^1^

Those words spoken by Alexander Graham Bell on March 10, 1876, have now led us to having an intercontinental world of possibilities and power in the palm of our hands. With worldwide smartphone penetration rate of >75%,^2^ communication via smartphone applications with instruction and images can assist with historically complicated medical dilemmas. A smartphone application (app) called WhatsApp (Meta Platforms, Mountain View, CA) serves as a medium to share photos, videos, texts, and voice memos. With this, there is great potential for mobile communication devices to use these visual images to improve care where language is a barrier.^4^ This app has also previously demonstrated to be a useful tool to assist physicians with patients in times of urgency.^3^ In this report, we present a patient case who presented with pacemaker battery depletion and then developed right sided phrenic nerve stimulation (PNS) after cardiac pulse generator exchange. Patient management was supported using WhatsApp. The patient provided written informed consent to share this study.

## Case report

**Figure 1:**
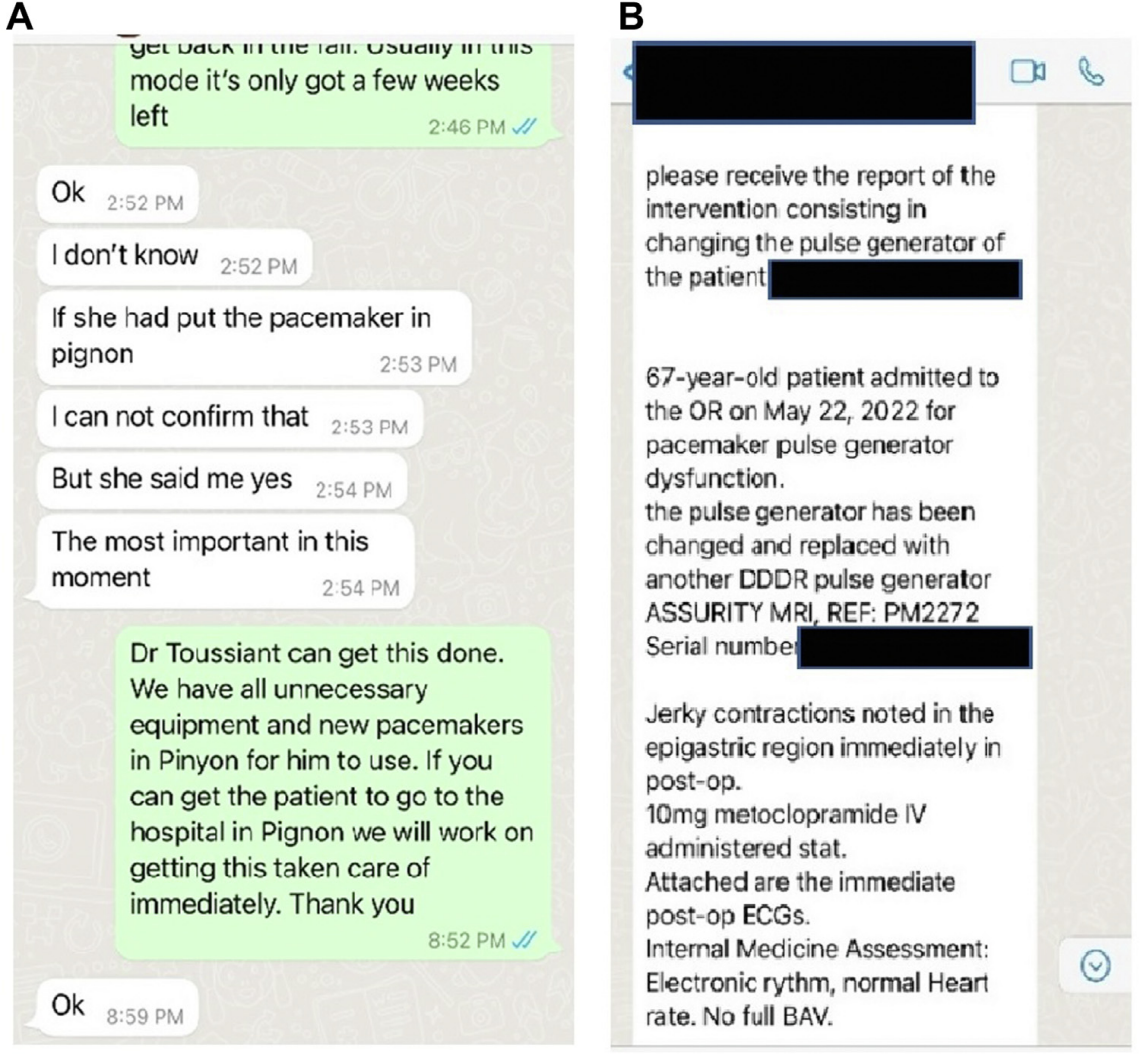
**A:** Communication of history and physical along with rhythm strips was shared across the WhatsApp platform between Haitian and American physicians. **B:** WhatsApp communication of postsurgery description of possible phrenic nerve stimulation.

A 67-year-old Haitian woman presented to the Hospital Bienfaisance de Pignon (a rural hospital in the northern plateau of central Haiti) with symptoms of fatigue and shortness of breath. She was found to have a heart rate of 50 beats/min and a slow ventricular paced rhythm by electrocardiogram (ECG) with atrioventricular dissociation. The patient was presumed to have underlying third-degree atrioventricular block. For over 10 years, this hospital has hosted a team of American cardiologists multiple times a year through Promise for Haiti.^7^ This program pairs cardiologists with a local, French Creole–speaking Haitian general surgeon to hold cardiology clinic and perform cardiac implantable electronic device insertions together. History and a physical exam with rhythm strips of the symptomatic patient were sent through WhatsApp to an American electrophysiologist in the United States for review (Figure 1A). It was determined that the unknown pacemaker battery had reached recommended replacement time and a pulse generator exchange was needed.

No cardiologists or electrophysiologists from Promise for Haiti were scheduled to be present in Haiti for 6 months. A decision to have the locally trained Haitian surgeon surgically change the pacemaker pulse generator was made. Immediately after a successful battery exchange (Abbott Assurity dual-chamber pacemaker [Abbott Vascular, Santa Clara, CA]), the patient started developing constant right-sided “epigastric jerky contractions” (Figure 1B). Video was sent through WhatsApp and appeared as right-sided PNS (Video 1).

**Figure 2:**
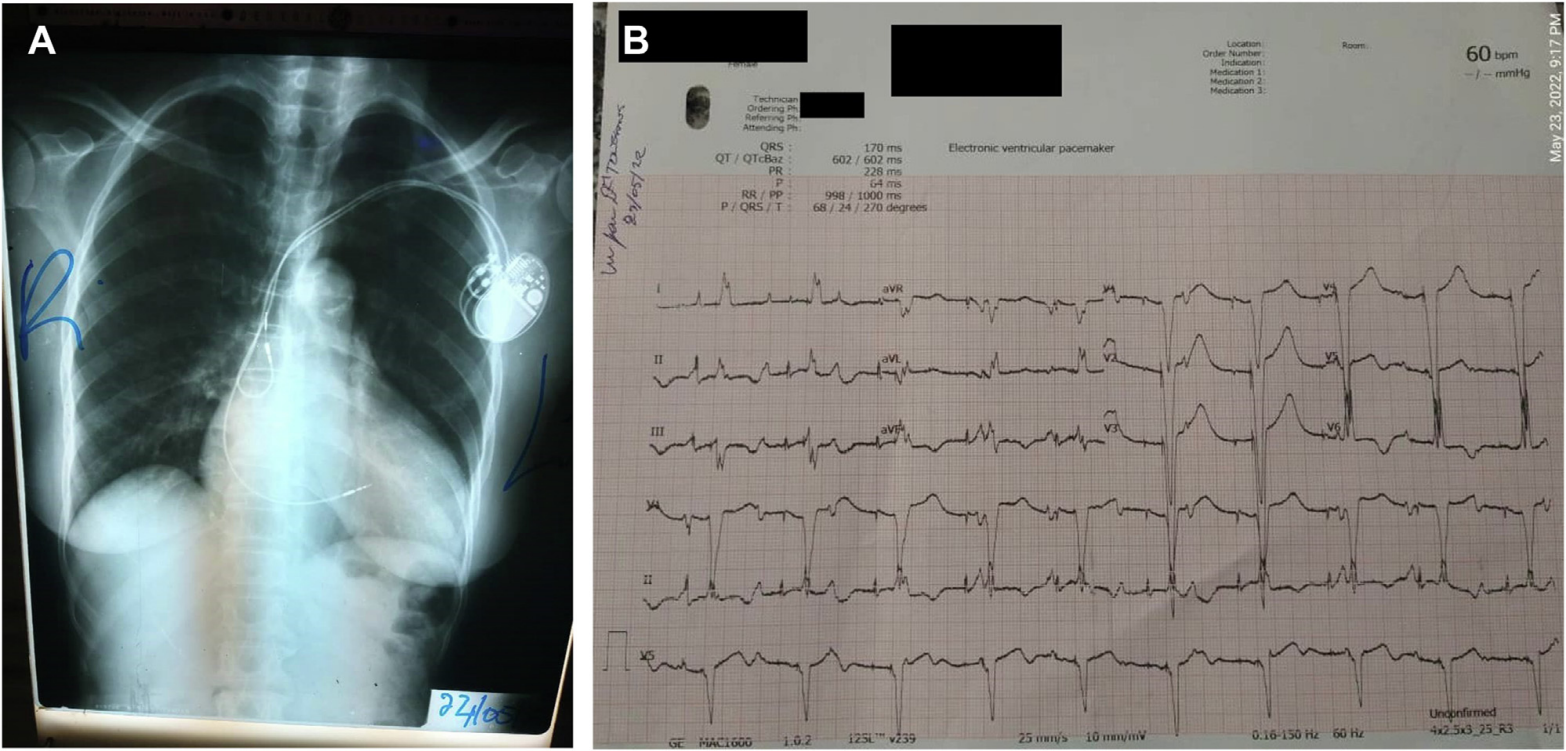
**A:** Chest radiograph showing acutely implanted pacemaker pulse generator with chronically implanted dual chamber pacing electrodes. The chronic atrial lead electrode tip appears to be positioned in the superior vena cava, with the tip electrode facing the lateral wall of the superior vena cava. **B:** Electrocardiogram showing undersensed P waves and noncapture in the atrial channel.

Chest radiographs and (ECG) were ordered, posted and viewed via WhatsApp*.* From examining the chest radiograph (Figure 2A), it was determined the patient’s chronic atrial lead was located in the superior vena cava with the tip electrode angled near the lateral wall of the superior vena cava. It is unknown when the chronic atrial lead was dislodged. We theorized that the atrial pacing electrode could potentially be stimulating the right phrenic nerve, thus contracting the right diaphragm muscle. The ECG demonstrated under sensing and noncapture in the atrium (Figure 2B).

**Figure 3:**
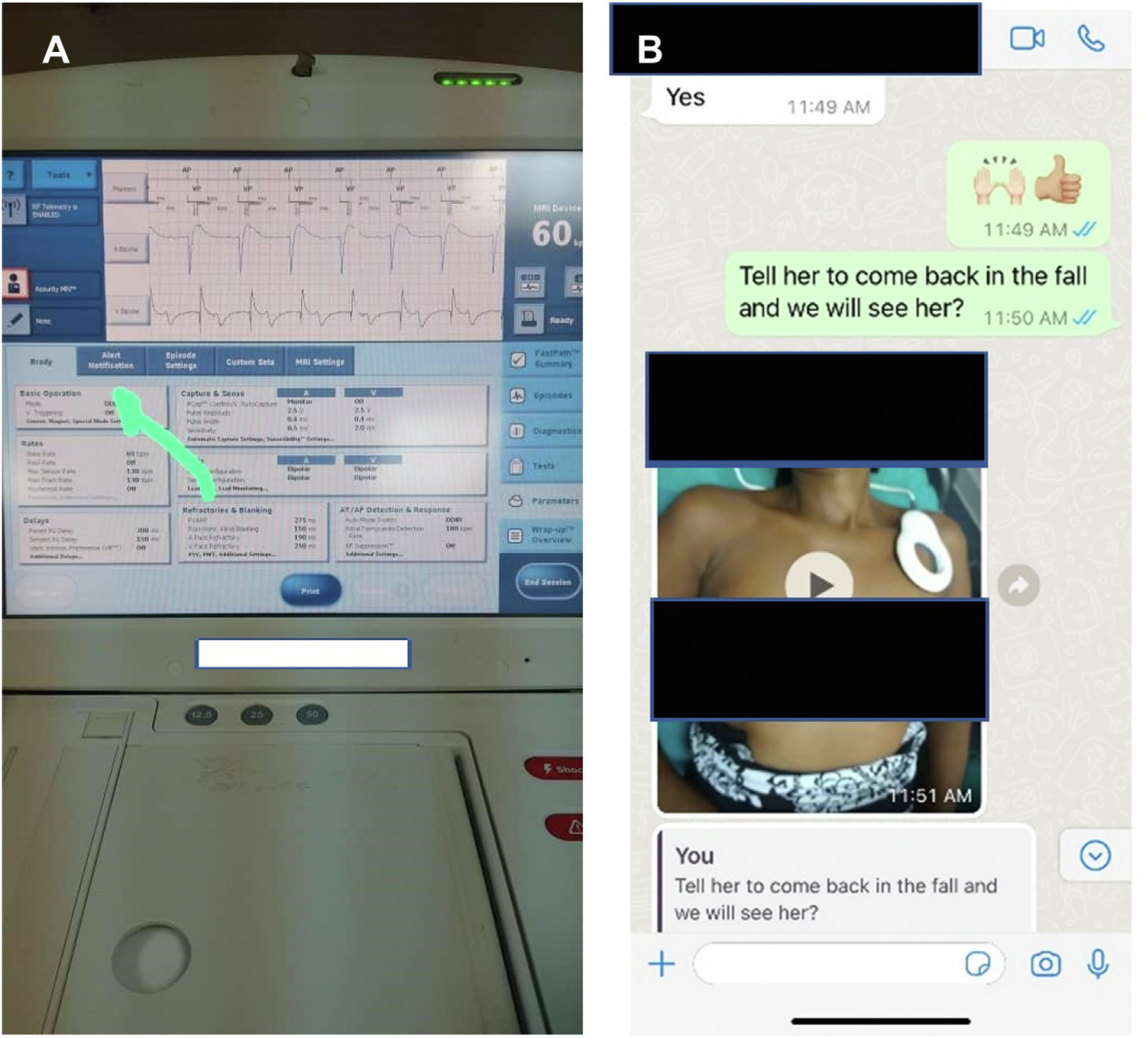
**A:** graphical user interface pacemaker programmer software screens with directional “touch here” arrows. **Center:** Pictures and videos were then sent back via WhatsApp illustrating confirmation of each step of the programming process until completion. **B:** Video confirming resolving of phrenic nerve stimulation.

With a nonfunctioning atrial lead potentially stimulating the phrenic nerve, a decision to program the pacemaker noninvasively via the Abbott Merlin programmer to VDD (atrial sense and ventricular pace/sense–only mode) was made. Using WhatsApp, a series of images of graphical user interface pacemaker programmer software screens with directional “touch here” arrows were sent to guide the local Haitian surgeon with reprogramming the pacemaker (Figure 3A). Pictures and videos were then sent back via WhatsApp illustrating confirmation of each step of the programming process till completion. Resolution of PNS and patient symptoms were sent in video form as well (Figure 3B).

## Discussion

We identified the use of a smartphone app, WhatsApp, which permitted immediate management of a patient with a pacemaker complication (PNS) following a generator replacement. PNS as a complication due to an implantable cardiac pacemaker was first reported by Dr James Sprinkle in 1963.^5^ The complication of pacemaker lead PNS can be uncomfortable and cause shortness of breath.^6^ The app offered the ability to perform programming of the pacemaker by using the app to send programmer images to a remote physician in Haiti. This is a significant advantage for the patient and pacemaker specialists who will not need to travel long distances to provide appropriate care and avoid exposure to infectious disease or adverse events. Significant challenges impede health care and utilization of current technology in developing countries. Consults between providers using WhatsApp has been well documented.^8^ Remote real-time programming for cardiac devices is evolving to improve patient lifespan and quality of life in areas previously impossible. Remote management of pacemakers via low-cost medium (WhatsApp) decreases the response time to troubleshoot device care and protects caregivers from travel associated infectious diseases, which continue to exist in underdeveloped tropical countries.^9^ In this case report, we demonstrate the feasibility of remote management of implantable pacemaker devices with existing communication infrastructure present in most developing countries (cellular phone service).

## Conclusion

Advances in smartphone apps can assist physicians and certified cardiac device specialist personnel in remote and medically underserved and socioeconomically disadvantaged communities. There is benefit in using pictures and graphics as well as video for disadvantaged medical personnel. We saw benefit in using pictures and graphics to help assist with programming a patient >1000 miles away providing an instant solution to PNS, which otherwise would not have been possible without time, travel, and expense. Without the ability to communicate and function through remote consultation, the PNS in this case would not have been resolved until experienced certified cardiac device specialist personnel could be physically present to reprogram the patient’s device, leading to significant morbidity for the patient. WhatsApp communication can assist with offering rapid specialist consults and troubleshooting of cardiac implantable electronic devices to underdeveloped rural areas.^8^

## Appendix. Supplementary data

Video 1 Phrenic Nerve video
